# Subinhibitory Concentration of Colistin Promotes the Conjugation Frequencies of *Mcr-1-* and *bla*_NDM-5_-Positive Plasmids

**DOI:** 10.1128/spectrum.02160-21

**Published:** 2022-03-01

**Authors:** Xia Xiao, Fuxin Zeng, Ruichao Li, Yuan Liu, Zhiqiang Wang

**Affiliations:** a College of Veterinary Medicine, Yangzhou University, Yangzhou, China; b Jiangsu Co-innovation Center for Prevention and Control of Important Animal Infectious Diseases and Zoonoses, Yangzhou, China; c Institute of Comparative Medicine, Yangzhou University, Yangzhou, China; d Institutes of Agricultural Science and Technology Development, Yangzhou, China; Forschungszentrum Jülich GmbH

**Keywords:** colistin, conjugation frequency, plasmid, horizontal gene transfer, ARGs

## Abstract

Horizontal gene transfer (HGT) plays a significant role in the spread of antibiotic resistance genes (ARGs). Most reported compounds promote HGT by increasing the cell membrane permeability. Colistin has been reported to increase the cell membrane permeability when exhibiting its antibacterial effect. Therefore, this study aimed to investigate the potential role of colistin in facilitating the dissemination of ARGs via plasmid conjugation by establishing an *in vitro* mating model. Three strains Escherichia coli (E. coli) DH5α, E. coli L65, and E. coli LD67-1 carrying plasmid RP4-7, *bla*_NDM-5_ positive IncX3 plasmid, and *mcr-1* positive IncI2 plasmid, respectively, were regarded as the donor strains and E. coli J53 as the recipient strain. Exposure to subinhibitory concentrations of colistin (1/4, 1/8, 1/16 MIC) significantly stimulated the conjugation frequency of RP-4 plasmid, wide-type IncI2 and IncX3 plasmid. Scanning electron microscopy revealed the shrunken cell membrane after colistin treatment, whereas propidium iodide dye and 1-*N*-Phenylnaphthylamine fluorescent probe showed the increased cell membrane permeability. Additionally, the expression level of the outer membrane proteins (*ompF* and *ompC*) was increased. These results indicate a break in the membrane barrier. The expression of the mating pair formation gene (*trbBp*) was promoted and the expression of the global regulatory genes (*korA*, *trbA*), which downregulates *trbBp* expression, was inhibited. Thus, the production of the mating pairing machine could be elevated after colistin exposure. These findings aid in understanding the hidden risks of colistin on the spread of antimicrobial resistance.

**IMPORTANCE** Antimicrobial resistance (AMR) dissemination is a growing global threat. As a last-resort treatment against multidrug-resistant and extensively drug-resistant Gram-negative bacteria, colistin has been used for prophylactic and therapeutic purposes in veterinary medicine. Previous studies have reported the presence of colistin residues in the intestinal tract and feces. The role of colistin in facilitating the conjugation frequency of *mcr-1-* and *bla*_NDM-5_-positive plasmids was confirmed in this study along with elucidating its potential mechanisms. This study raises awareness of the potential AMR dissemination roles induced by colistin in environmental settings.

## INTRODUCTION

Antimicrobial resistance (AMR) is a growing global health threat. AMR has been predicted to have a mortality rate of 10 million, including a cumulative 100 trillion dollars in financial costs, by 2050 (1). The dissemination of AMR is mainly attributed to mutations, vertical gene transfer (VGT) and horizontal gene transfer (HGT), with HGT playing a major role in the environmental spread of antibiotic resistance genes (ARGs) (2). There are mainly three pathways for HGT: transformation, transduction, and conjugation (3). Conjugation, which transfers DNA by direct physical cell-to-cell contact via a pilin bridge or pore channel, is the most dominant HGT pathway (4, 5). Plasmid, especially transferable plasmid, is the major vehicle of conjugation. Generally, spontaneous frequency of plasmid conjugation is low (6); however, various compounds such as nanomaterials, disinfectants, disinfection by-products, ionic liquids, heavy metal, anticonvulsants, antiepileptic, and nonnutritive sweeteners, have been reported to promote plasmid conjugation rate (7–13). The underlying mechanisms include increased reactive oxygen species (ROS) level, SOS response activation, enhanced cell membrane permeability and pilus generation (11, 14–16).

Plasmid-encoded ARGs have been commonly speculated to pose a metabolic burden on the host and their evolutionary success and maintenance depend on positive selection, which is mainly antibiotics. Thus, sublethal levels of antibiotic exposure have contributed to the spread of antibiotic resistance through positive selection and promoted the fitness of plasmids. Moreover, various antibiotics such as tetracycline, sulfamethoxazole, gentamicin, imipenem, azithromycin and quinolones have been found to enhance the conjugation frequency of integrating conjugative elements (ICEs) (17–20). However, the effect of polypeptide antibiotic colistin on promoting plasmid conjugation requires further investigation.

Colistin is a secondary metabolite of the Gram-positive soil bacterium *Paenobacillus polymyxa* subsp. *Colistinmus* (21). For decades, colistin has been used in veterinary medicine for prophylactic and therapeutic purposes (22). Although the use of feed additives was forbidden in China, the oral administration of colistin via drinking water was widely prevalent in the livestock and poultry industries for therapeutic or metaphylaxis purposes (23), with high residues present in the intestinal tract and feces (24–26). Moreover, due to the emergence of multidrug-resistant (MDR) and extensively drug-resistant (XDR) Gram-negative superbugs, it has been used as a last-resort treatment against MDR and XDR Gram-negative bacteria (27, 28). It functions by killing bacteria and has a synergistic effect with several antibiotics by increasing the cell envelope permeability and thereby inducing cellular content leakage (29, 30). As enhanced cell membrane permeability is one of the underlying mechanisms by which compounds promote plasmid conjugation; therefore, colistin was speculated to impact plasmid conjugation.

This study aims to verify the hypothesis that the subinhibitory concentration of colistin plays a role in facilitating the dissemination of ARGs via plasmid conjugation. The RP4-7 plasmid, *bla*_NDM-5_-positive IncX3 plasmid and *mcr-1*-positive IncI2 plasmid were used to investigate whether colistin promoted plasmid-mediated conjugative transfer. The underlying mechanisms of conjugative transfer were inferred from cell membrane permeability, ROS production and conjugation-related gene expression.

## RESULTS

### MIC determination.

**FIG 1:**
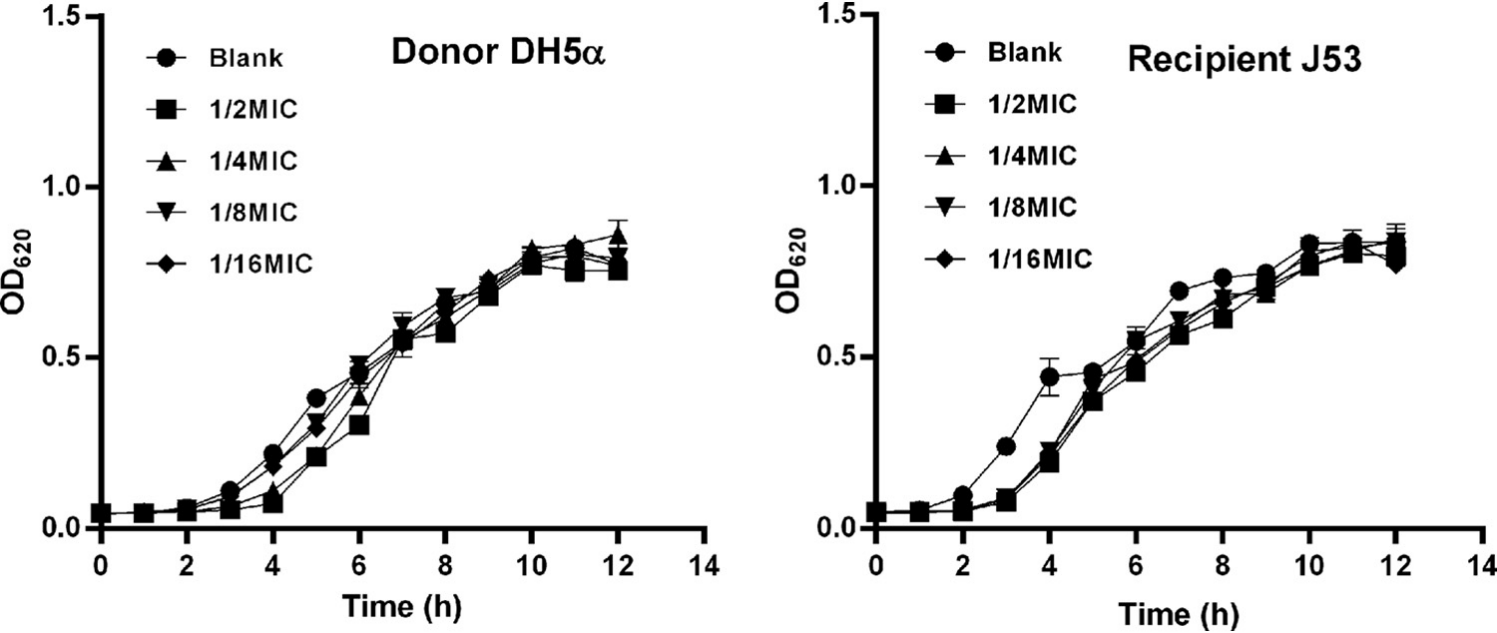
The impact of colistin on the growth of the donor (DH5α) and recipient (J53) strains at different sub-inhibitory concentrations.

The MICs of colistin against DH5α, J53, L65 and LD67-1 were 0.125, 0.5, 1 and 4 mg/L, respectively. The MIC values of the donor and recipient strains were different; therefore, the lowest MIC of colistin was used when applying colistin to the conjugation transfer system. There was no difference in DH5α growth between the colistin treated groups and blank control, whereas the growth of J53 was slightly inhibited in the colistin treated groups during the first 6 h. However, the subinhibitory concentrations of colistin did not affect the growth of the donor and recipient strains at 12 h (Fig. 1). Thus, the impact of the subinhibitory concentration of colistin on bacterial growth was neglected at 12 h.

### Subinhibitory concentration of colistin increase the CTF of plasmid.

**FIG 2:**
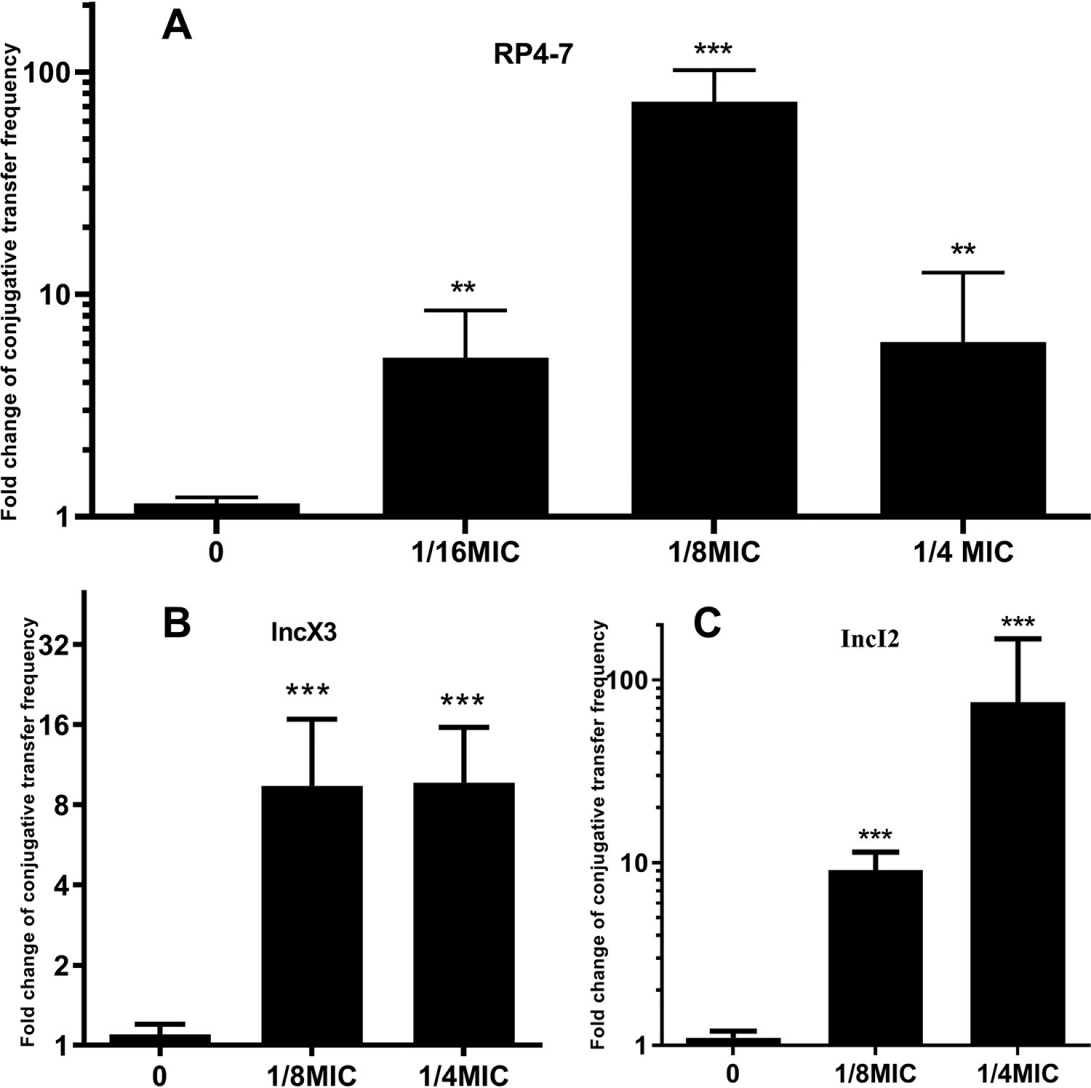
Colistin facilitates the conjugation frequencies of RP4-7 plasmid, IncI2 plasmid and IncX3 plasmid. (A) The conjugation frequency of RP4-7 was promoted in a concentration independence way under the exposure of sub-inhibitory concentration of colistin; (B) the conjugation frequency of *bla*_NDM-5_ positive IncX3 plasmid was elevated under the exposure of sub-inhibitory concentrations of colistin; (C) the conjugation frequency of *mcr-1* positive IncI2 plasmid was promoted when exposed to 1/8MIC and 1/4MIC of colistin. Significant differences between colistin treated groups and the blank control (drug free) were tested with using one-way analysis of variance: **, *P* < 0.01, ***, *P* < 0.001.

As shown in Fig. 2A, colistin facilitates the CTF of the RP4-7 plasmid at different concentrations but not in a concentration-dependent manner. At 1/8 MIC, the CTF of RP4-7 reached a peak of 9.84 × 10^−4^, which was 38 times higher than that of the antibiotic-free group.

To determine if colistin can facilitate the CTF of wide-type plasmids and understand the clinical impact of colistin on CTF, the effects of colistin on the CTF of wild-type plasmid, IncI2 plasmid (carrying *mcr-1* gene) and IncX3 plasmid (carrying *bla*_NDM-5_ gene) were determined. The results showed that the CTF of the IncI2 plasmid and IncX3 plasmid was significantly increased. by approximately 8-fold (Fig. 2B). and 10-fold (Fig. 2C), respectively, at both 1/4 and 1/8 MICs. This indicates that the subinhibitory concentration of colistin promotes the CTF of various plasmids that carry important resistance genes.

### Effect of colistin on the penetration of the cell membrane.

**FIG 3:**
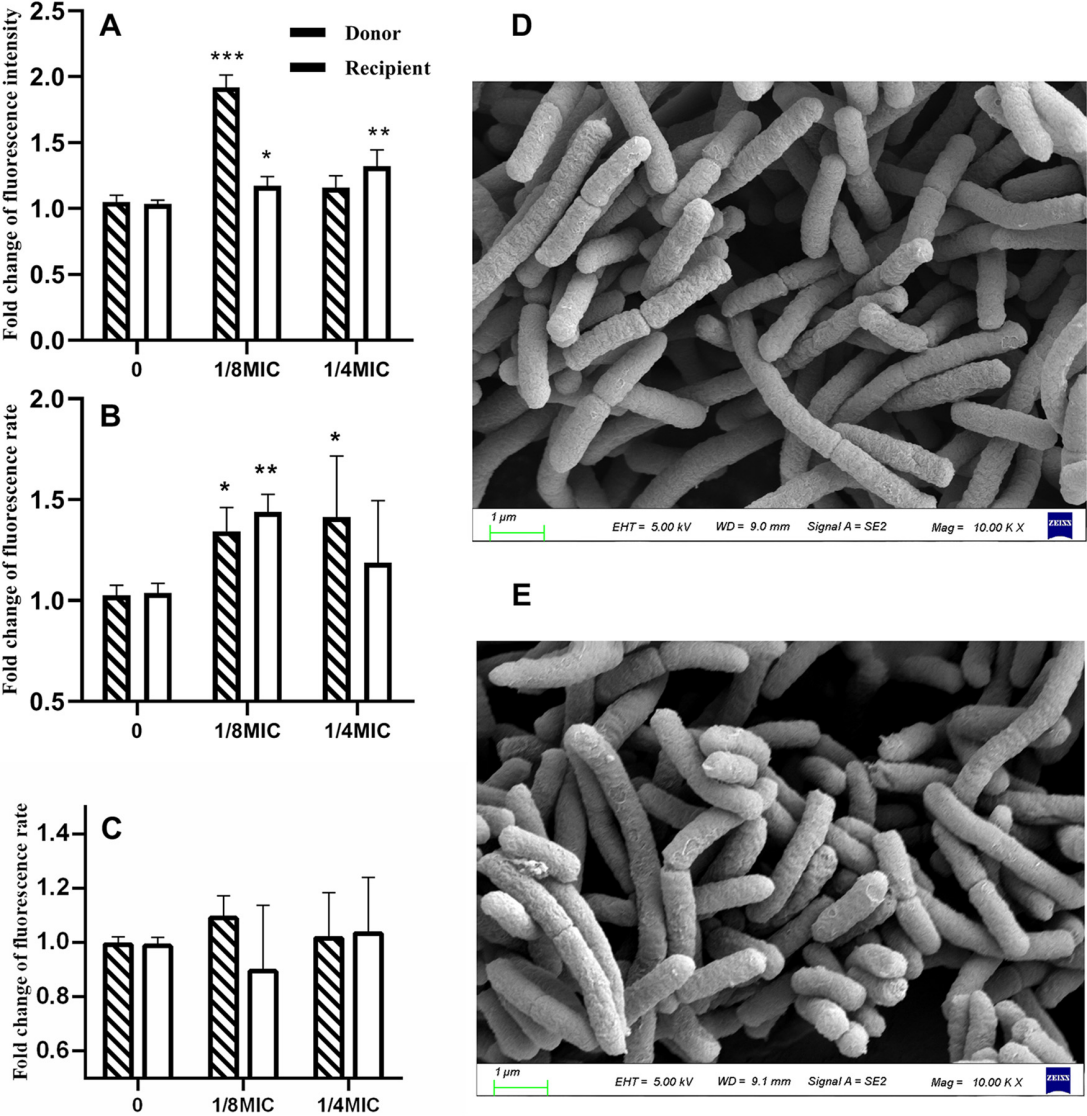
Subinhibitory concentrations of colistin induced significant changes in the cell membrane permeability and bacterial membrane barrier but not on reactive oxidative species (ROS) production. (A) Fold changes in the outer membrane permeability using the 1-*N*-Phenylnaphthylamine (NPN) probe on exposure to subinhibitory colistin concentrations; (B) fold changes in the inner membrane permeability using the propidium iodide (PI) probe on exposure to subinhibitory colistin concentrations; (C) fold changes in ROS generation on exposure to subinhibitory colistin concentrations; the surface morphology observed using scanning electron microscope in the absence (D) and presence (E) of 1/8 MIC of colistin. Significant differences between the colistin treated groups and the blank control (drug-free) were determined using one-way analysis of variance: *, *P* < 0.05, **, *P* < 0.01, ***, *P* < 0.001.

The conjugation process is dependent on the cell membrane, and colistin induces bacterial death by destroying the cell envelope and causing cellular content leakage. In order to elucidating the potential biological mechanisms of conjugative transfer, this study determine if the subinhibitory concentration of colistin could increase the permeability of the cell membrane. Thus, membrane permeability related biochemical parameters, including outer membrane permeability, inner membrane permeability and ROS generation, were evaluated. After exposure to the subinhibitory concentrations of colistin (1/8 MIC, 1/4 MIC), the fluorescence of NPN-probed cells was enhanced in the donor and recipient strains (Fig. 3A). Similarly, the fluorescence of PI-probed cells was enhanced in both strains after exposure to 1/8 MIC and 1/4 MIC of colistin (Fig. 3B). This demonstrates that colistin disrupted the integrity of the bacterial outer membrane and cytoplasmic membrane. Further, the evaluation of ROS accumulation in the donor and recipient strains after exposure to colistin showed no significant increase in fluorescence between the colistin-treated and blank control groups (Fig. 3C). Finally, visualization of the cell membrane changes using SEM showed local damages on the bacterial surface after colistin treatment (Fig. 3E).

### Effect of colistin on the expression of conjugation-related genes.

**FIG 4:**
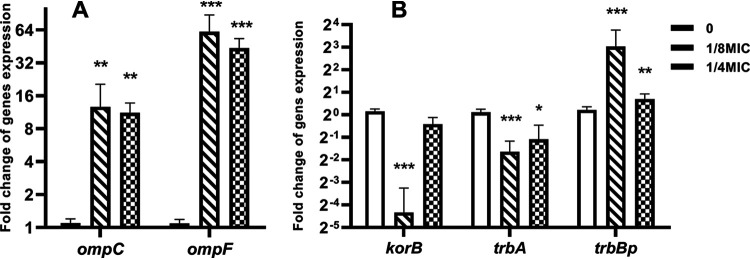
Effect of colistin on the expression levels of pore forming and conjugation-related genes. (A) Fold changes in the relative expression levels (mRNA) of the cell membrane-related genes in the donor and recipient mixture (1:1) on exposure to subinhibitory colistin concentrations; (B) fold changes in the relative expression levels of the conjugation-related genes in the donor and recipient mixture (1:1) on exposure to subinhibitory colistin concentrations. The expression of the global regulatory genes (*korB*, *trbA*) was inhibited, while the expression of the mating pair formation gene (*trbBp*) was promoted. Significant differences between the colistin treated groups and the blank control (drug-free) were determined using one-way analysis of variance: *, *P* < 0.05, **, *P* < 0.01 ***, *P* < 0.001.

To explore the underlying mechanisms, qPCR was performed to analyze the changes in the expression of conjugation-related genes. OmpC and OmpF play a key role in the pore or channel formation and membrane transport and are vital for efficient mating pair formation during conjugation. The expression levels of *ompC* and *ompF* in colistin treated cells were more than 10 times that in control cells (Fig. 4A). The upregulated expression of *ompC* and *ompF* was consistent with the increase in cell membrane permeability, which was detected using fluorescent probes.

Meanwhile, determining the expression of the *trbBp* gene, which is involved in the mating pair formation for conjugation, and the global regulation genes (*korB*, *trbA*), which inhibit the expression of the *trbBp* gene, (Fig. 4B) revealed the downregulated expression of *korB* and *trbA* genes on colistin treatment at both subinhibitory concentrations, except for *korB* at 1/4 MIC. The expression level of *trbBp* showed a significant and slight upregulated trend in 1/8 MIC and 1/4 MIC colistin treated cells. The expression levels of the other tested genes showed no significant differences between the colistin treated and control groups except for *traG* at 1/8 MIC (Fig. 4B, Fig. S1). Therefore, colistin has the potential to promote plasmid CTF by inhibiting the expression of the global regulator genes *korB* and *trbA* and upregulating the expression of the mating pair formation gene *trbBp*.

## DISCUSSION

Colistin is currently used as a last-resort treatment against MDR and XDR Gram-negative bacteria in clinical settings. Concerns regarding its ability in inducing plasmid-mediated resistance genes at sub-lethal concentrations have been raised. However, the effect of colistin on the spread of antibiotic resistance through HGT, especially plasmid conjugation, remains unexplored to the best of our knowledge. This study illustrates that the conjugation frequency of the RP4-7 and wide-type plasmids bearing important resistance genes could be significantly stimulated on colistin exposure at sub-MICs.

The discovery and development of mobilized colistin resistance genes (*mcr-1* and its variants) led to the restriction of its use as a feed-additive in many countries, such as China (31, 32). However, its oral administration via drinking water in the livestock and poultry industries are widely used for therapeutic or metaphylaxis purposes (23). This form of administration and low absorption through the gastrointestinal tract usually leads to whole-flock treatment. Additionally, colistin concentration in the small intestine of chicken was reported to be more than 1.1 mg/kg for more than 76 h after administration with the current clinical dose (2.5-mg colistin base equivalent/kg/d) (24). The residue of colistin in the gastrointestinal tract might not only pose a selective pressure to less susceptible isolates present within the intestinal tract but also promote the plasmid conjugation and ARGs horizontal dissemination. Furthermore, trace concentrations of the drug were found in the excreted feces. Xia et al. reported that colistin was detected in feed and fresh manure samples at 67 mg/kg and 17 mg/kg, respectively, in 2016 (25). Rasschaert et al. also detected colistin residue in pig manure (26), which is a repository of ARGs. This suggests that the colistin residue in the manure might facilitate the HGT of ARGs and threaten the effectiveness of antimicrobials. Therefore, the fate of colistin in feces and manure and its effect on conjugation should be monitored in future. A study reported colistin A and colistin B in 16 out of 54 feed samples (29.63%), with the highest concentration at 11,928 and 9,999 μg/kg (33), which calls for stricter surveillance of colistin contamination.

Elucidating the potential mechanisms of colistin in the enhancement of the conjugative transfer of the RP4-7 plasmid revealed two factors that play key roles in the conjugation process, including the increased permeability of cell membranes and the increased conjugation activity of the RP4-7 plasmid.

The outer and inner cell membrane permeability increased significantly based on the NPN and PI stain. Additionally, SEM observation showed that the membrane barrier was impaired. Several reports have confirmed that the increased permeability of the cell membrane and impairment of the cell membrane promotes the conjugation frequency (10, 11). Thus, colistin facilitates the conjugative transfer of plasmids by increasing the permeability of cell membranes. Compounds such as the antiepileptic drug carbamazepine and triclosan have been reported to promote the CTF of plasmid by increasing ROS production (10, 11). However, the ROS generated by the cells in this study showed no significant difference between the colistin and control groups.

OmpC and OmpF play a key role in pore formation and membrane transport (34, 35). The significant upregulation in *ompC* and *ompF* transcriptional levels during colistin exposure could promote the transport of plasmid DNA and thereby facilitate plasmid conjugation. Therefore, colistin enhancement in conjugative plasmid transfer could be regulated via the *omp* genes. The expression of *trbBp* was significantly upregulated on colistin exposure, whereas the expressions of *korB* and *trbA* were downregulated. The KorB and TrbA proteins are key regulators of the plasmid genes required for conjugative transfer, which act by repressing the transcription of *trbBp* (36). The *trbBp* gene is the primary promoter responsible for the expression of mating pair formation genes (37). This suggests that colistin facilitates the mating pair formation by inhibiting the expression of regulatory genes (*korB*, *trbA*) and accelerating the expression of *trbBp*. Therefore, colistin has the potential to promote CTF by regulating mating pair formation, which subsequently could increase the transfer of plasmids.

Though some sub-MIC antibiotics have been reported to stimulate horizontal transfer of antibiotic resistance (17), this paper first found that Polypeptide antibiotics colistin promote the horizontal gene transfer of RP4 plasmid and clinical important plasmids. The underline possible mechanism of this phenomenon was uncovered (increasing the permeability of the bacterial cell membrane and promoting the production of the mating pairing machine). The result was a well strong evidence to confirm the viewpoint that sub-MIC of antibiotics stimulate horizontal transfer of antibiotic resistance. However, this study has certain limitations. Although the effect of colistin in the conjugation transfer of the wide-type plasmids between intragenera was confirmed, intergenera verification was not performed. Additionally, the conjugative transfer of ARGs occurring in the environment is complex. Future studies should focus on the impact of colistin on the conjugative transfer of ARGs within complex communities, for example, in feces manure, gut microbiota, wastewater and soil.

In conclusion, the role of the subinhibitory concentration of colistin in promoting plasmid transfer frequency was confirmed using RP4-7 plasmid, *bla*_NDM-5_-positive IncX3 plasmid and *mcr-1*-positive IncI2 plasmid. Colistin has the potential to facilitate this function by increasing membrane permeability, stimulating pore formation and mating pair formation. These findings improve our awareness of the hidden risks of colistin on the spread of ARGs.

## MATERIALS AND METHODS

### Bacterial strains and antibiotics.

Escherichia coli DH5α strain harboring an RP4-7 plasmid was provided by College of Bioscience and Biotechnology, Yangzhou University. E. coli DH5α (DH5α, with the RP4-7 plasmid carrying the chloramphenicol [CHL] and ampicillin [AMP] resistance genes) served as the donor, whereas E. coli J53 served as the recipient (Sodium Azide _[NaN3]_ resistant). E. coli L65 bearing the *bla*_NDM-5_-positive IncX3 plasmid was isolated from goose feces, whereas E. coli LD67-1 bearing the *mcr-1-*positive IncI2 plasmid was isolated from elk feces. Both isolates were attributed to our laboratory and used as donor strains for the conjugation assay. The basic information of these strains is listed in Table 1. Colistin was purchased from Yuanye Biological Technology Company (Shanghai, China). Other analytical reagents were purchased from Sigma-Aldrich (USA) and Beyotime biotechnology company (Shanghai, China).

**TABLE 1:** Basic information of strains used in the conjugation experiment

Strains	MLST types	Resistance genes	Plasmid names	No. of resistance genes	Accession no. (NCBI)
L65	ST3076	*tet*(A), *qnrS1, dfrA14, folR,* *bla*_TEM-1C_, *bla***_NDM-5_**	pL65-2(IncFIB, IncFII) pL65-9(IncX3)^a^	6 1	CP034739 CP034744
LD67-1	ST1485	*mcr-1*, *bla***_CTX-M-132_** *aadA1, dfrA14, tet*(A), *qnrS1, ARR-2*, *bla***_OXA-10_**, *cmlA1, floR*	pLD67-1-MCR1( IncI2)^b^ pLD67-1-157kb (IncHI2, IncHI2A)	2 8	CP061186 CP061187

### MIC determination.

The MIC of colistin against DH5α, J53, L65 and LD67-1 was determined using the broth microdilution method according to the Clinical and Laboratory Standards Institute (CLSI) guidelines and interpreted following the CLSI standard (38). E. coli ATCC 25922 was used as the quality control strain.

### Bacterial growth of donor and recipient strains.

Overnight cultures of DH5α and J53 were diluted with fresh Luria-Bertani (LB)-broth to the optical density (OD600) of 0.5. Colistin was added to each bacterium to attain a final concentration of 1/16 MIC, 1/8 MIC, 1/4 MIC and 1/2 MIC. A sample without colistin was used as the control in each experiment. The mixtures were incubated at 37°C. The OD600 was measured every 1 h for 12 h. Each experiment was performed in triplicates.

### *In vitro* conjugative transfer system.

A conjugation experiment was performed using phosphate-buffered saline (1 × PBS, pH = 7.2) based on the study by Wang et al., with some modifications (39). Briefly, the donor (DH5α) and recipient (J53) strains were cultivated overnight in liquid LB at 37°C. Following this, the strains were centrifuged for 5 min at 6000 × *g* in 4°C. The supernatants were discarded, and the pellets were washed twice using PBS. The pellets were resuspended in PBS to obtain a final bacterial density of 1 × 10^8^ CFU (CFU)/mL. The donor and recipient strains were mixed at a ratio of 1:1 and exposed to a series of colistin concentrations (1/16MIC, 1/8MICand 1/4 MIC). A sample without colistin was used as the control.

After incubation at 30°C for 12 h with constant shaking at 160 rpm, 50 μL of the mixtures was spread onto LB agar plates containing 20-mg/L CHL, 100-mg/L AMP and 200-mg/L NaN_3_ and incubated for 48 h to determine the number of transconjugants. The total number of recipient strains was determined by plating the diluted mating mixtures onto LB agar plates containing 200-mg/L NaN_3_. The conjugation transferring frequency (CTF) was determined using the manual CFU counting of transconjugants (CFU/mL) per number of recipients (CFU/mL). Representative colonies from conjugants were confirmed using antimicrobial susceptibility testing. All conjugative mating experiments were performed in biological triplicates.

### The impact of colistin on the distribution of important wide-type plasmids.

To determine the impact of colistin on the CTF of wide-type plasmid, two widespread plasmids harboring an important resistance gene (*mcr-1-*positive IncI2 plasmid and *bla*_NDM-5_-positive IncX3 plasmid) were used. A conjugation experiment was conducted following the same procedure as plasmid RP4-7 using the 1/8 MIC and 1/4 MIC colistin concentrations. The LB agar plates containing 200-mg/L NaN_3_ and meropenem (2 mg/L) or 200-mg/L NaN_3_ and colistin (1 mg/L) were used to select transconjugants. Representative colonies of conjugants were confirmed using PCR (PCR) (*bla*_NDM_ primer F-5’CT TCCAACGGTTTGATCGTC3’/R-5’ATTGGCATAAGTCGCAATCC3’; *mcr-1* primer F-5’AG ATCCTTGGTCTCGGCTTG3’/R-5’AGTCCGTTTGTTCTTGTGGC3’) and antimicrobial susceptibility testing.

### Outer membrane permeability assay.

E. coli DH5α and J53 (10^6^ CFU/mL) was treated with 0, 1/8 MIC and 1/4 MIC colistin for 12 h. The cells were washed twice using PBS and followed by fluorescent probe 1-*N*-Phenylnaphthylamine (NPN) (10 μM) addition to evaluate the outer membrane integrity. After shaking at 100 rpm for 30 min in the dark, the fluorescence intensity was measured using a Microplate System (Biotek Synergy 2) at an excitation wavelength of 350 nm and an emission wavelength of 420 nm.

### Inner membrane permeability assay.

The fluorescent dye propidium iodide (PI) (30 μM) was added to the same cells to perform the outer membrane permeability assay. After shaking at 100 rpm for 30 min in the dark, fluorescence was measured using a CytExpert Flow Cytometer (Beckman, USA) at an excitation wavelength of 488 nm and an emission wavelength of 630 nm. The heat-treated (2 h at 80°C) cells were used as the control for the inner membrane damaged cells. All tests were performed using biological triplicates. The results were analyzed using the CytExpert 2.0 software (Beckman, USA).

### Total ROS assay.

The 2′,7′-dichlorofluorescein diacetate (DCF-DA) dye was used to determine intracellular ROS generation. Briefly, DCF-DA (100 μM) was added to the donor E. coli DH5α and recipient E. coli J53 suspensions (in PBS at approximately 10^6^ CFU/mL). After shaking at 100 rpm for 30 min in the dark, the cells were washed twice using PBS to remove excess probes. Subsequently, antibiotics at subinhibitory concentrations (0, 1/8 MIC and 1/4 MIC) were added to the bacterial cells, which were incubated for 2 h at room temperature in the dark. Bacterial cell fluorescence was measured using a CytExpert Flow Cytometer (Beckman, USA) at an excitation wavelength of 488 nm and an emission wavelength of 630 nm.

### The surface morphology observed using a scanning electron microscope (SEM).

E. coli DH5α and J53 (10^8^ CFU/mL) were treated with 0 and 1/8 MIC colistin for 12 h. After washing with PBS, the pellets were resuspended gently with precooled 2.5% (vol/vol) glutaraldehyde for 24 h. The fixed strains were washed thrice with PBS (10 min each). Subsequently, the samples were dehydrated using an ethanol gradient method, which included 100% ethanol and sodium sulfate, for 15 min. The samples were dried using the critical point dryer and subsequently treated with spray-gold and observed under a Gemini SEM 300 (Carl Zeiss, Germany).

### mRNA expression of conjugative transfer-related genes.

To mimic real conditions, the expression levels of porin genes and conjugation related genes in the mixture of donor and recipient bacteria (1:1 ratio at a ensity of 1 × 10^8^ CFU/mL) treated with sub-MIC colistin at 30°C for 12 h were investigated (10). The EASYspin bacterial RNeasy minikit was used to extract total RNA. The extracted RNA was reverse transcribed into cDNA using a PrimeScript RT reagent kit (TaKaRa, Japan). Real-time quantitative PCR (qPCR) was used to quantify gene expression using SYBR Premix *Ex Taq* *Ex Taq* II (TaKaRa, Japan). The expression levels of porin forming related genes: *ompF*, *ompC;* ROS related genes: *sodA*, *sodC*; mating pair formation genes: *korA*, *korB*, *trbA*, *traF*, *trbBp* and *traJ* were determined. *16S rRNA* was used as the internal control. Primer sequences are listed in Table S1. The qPCR primers were synthesized by Qinke Biotech Co., Ltd. (Nanjing, China), and qPCR was performed using a Mini Opticon real-time PCR system.

### Statistical analyses.

GraphPad Prism 8.2.1 was used for data analyses. Data are expressed as mean ± standard deviation. Significant differences were assessed using a one-way analysis of variance, with *P* < 0.05 being considered statistically significant.

### Data availability. 

The data were available at the corresponding author.
