# Supplementary material for: Subinhibitory Concentration of Colistin Promotes the Conjugation Frequencies of Mcr-1- and blaNDM-5-Positive Plasmids
Source: Microbiol Spectr. 2022 Mar 1;10(2):e02160-21. doi: 10.1128/spectrum.02160-21 (PMC9045390; doi:10.1128/spectrum.02160-21)
Supplement: SUPPLEMENTAL FILE 1 — Supplemental material. Download SPECTRUM02160-21_Supp_1_seq1.pdf, PDF file, 0.2 MB [file spectrum02160-21_supp_1_seq1.pdf]

Table S1 The Primer sequences and product size

Genes	Primer	Sequence of primer(5'-3')	Product Size /bp
ompC	*ompC*-F	AAGTAGTAGGTAGCACCAACATCA	163
	ompC-R	GGGCGAACAAAGCACAGAA	
ompF	*ompF*-F	GGTCTGCGTCCGTCCAT	99
	ompF-R	GGTTGCGCCCACTTCA	
korA	*korA*-F	TCGGGCAAGTTCTTGTCC	147
	korA-R	GCAGCAGACCATCGAGATA	
korB	*korB*-F	CTGGTCGGCTTCGTTGTA	149
	korB-R	TGAAGTCACCCATTTCGGT	
trbA	*trbA*-F	TGGAAACTCCCCTACCTCTT	120
	trbA-R	CCACACTGATGCGTTCGTAT	
trbBp	*trbBp*-F	CGCGGTCGCCATCTTCACG	131
	trbBp-R	TGCCCGAGCCAGTACCGCCAATG	
traG	*traG*-F	TACGCTGATTGGCGACAGTT	119
	traG-R	ACGTGCTGCATAACTGGTGA	
traF	*traF*-F	TGTTCTTTTACCGGGGGCAG	132
	traF-R	AATCCGGCAACAGCGGATTA	
traJ	*traJ*-F	TGGAAGCATACAGGAATGAGCA	153
	traJ-R	TGACGAACATGAGCAGCATC	
sodA	*sodA-F*	GAAAGGCGATAAACTGGCGG	75
	sodA-R	GCGCCAGAAATAGCTTCACC	
sodC	*sodC-F*	GCGCCTCGTCTGAAATCACT	127
	sodC-R	TTACACCACAGGCATAGCGT	
16sRNA	*16sRNA*-F	CCTACGGGAGGCAGCAG	194
	16sRNA-R	ATTACCGCGGCTGCTGG	

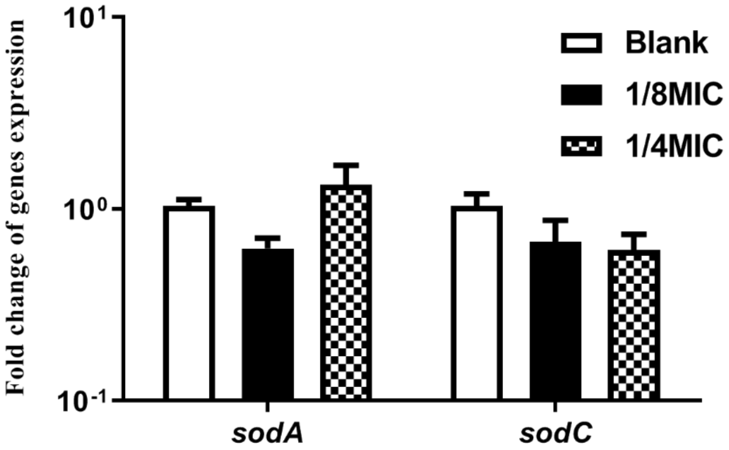

Figure S1. Effect of colistin on the expression levels of ROS related genes. Fold changes in the relative expression levels (mRNA) of the ROS-related genes in the donor and recipient mixture (1:1) on exposure to sub-inhibitory colistin concentrations.
